# Evaluation of safety, comfort, and design ergonomics in ambulance retrieval vehicles: a survey in Southern India

**DOI:** 10.1186/s12245-026-01316-9

**Published:** 2026-07-31

**Authors:** Lavanya Aradhya, Vrinda Lath, Freston Marc Sirur, Jayaraj Mymbilly Balakrishnan, Arundhathi Hebbar, Aniketh Kumar Shetty

**Affiliations:** 1 Higher Colleges of Technology, Abu Dhabi, UAE; 2 Department of Emergency Medicine, Kasturba Medical College, Manipal Academy of Higher Education, Manipal, India; 3 Department of Emergency Medicine, Kasturba Medical College, Manipal Academy of Higher Education, Manipal, India; 4 Emergency Medical Technology, Manipal College of Health Professions, Manipal Academy of Higher Education, Manipal, India; 5 Department of Anesthesiology, Kasturba Medical College, Mangalore, Manipal Academy of Higher Education, Manipal, India

**Keywords:** Emergency medical services, Ambulances, Ergonomics, Emergency medical technicians, Occupational health, Patient safety, Prehospital care, Ambulance design

## Abstract

**Background:**

Ambulance interior design influences the safety, comfort, and operational efficiency of Emergency Medical Services (EMS) personnel. However, evidence regarding ergonomic challenges faced by EMS providers in the Indian prehospital setting remains limited.

**Objective:**

To evaluate EMS personnel perspectives on ambulance ergonomics, safety, comfort, and factors affecting patient care during transport.

**Methods:**

A multicentre, cross-sectional survey-based observational study was conducted among Emergency Medical Technologists (EMTs) and paramedics, recruited by convenience sampling, who were involved in patient transport using standard Advanced Life Support (ALS) ambulances across Southern India. Data on ergonomic challenges, safety concerns, occupational injuries, and patient-care factors were collected using a questionnaire developed from existing literature and refined through focused-group discussion and expert content review. Data were analysed using descriptive statistics and chi-square tests in jamovi.

**Results:**

A total of 130 EMS providers participated. Limited patient-cabin space (60%) and difficulty accessing equipment (50%) were the most frequently reported factors affecting safety and comfort. Half of the respondents reported concerns regarding EMT seat positioning during patient care. Uneven roads (49.23%) and sudden vehicle breakdowns (41.53%) were the leading causes of transport-related injuries. CPR was the procedure most commonly associated with injuries to the care giver (53.84%), while back pain was the predominant occupational health hazard (57.69%). Significant associations were observed between work experience and perceptions of ambulance ergonomics, injury mechanisms, patient safety concerns, and occupational health hazards (*p* < 0.05).

**Conclusion:**

EMS personnel reported substantial concerns regarding ambulance ergonomics, particularly seat positioning, equipment accessibility, cabin workspace, and occupational safety. These findings emphasize the importance of further ergonomic research in ambulance design to enhance operational ease during in transit care while assuring provider and patient safety.

## Introduction

Emergency Medical Services (EMS) is an evolving field focused on the transport and prehospital management of critically ill patients. Global efforts under WHO’s 13th General Programme of Work (2019–2023) have supported the development of organized emergency care systems as part of universal health coverage [1]. The experience of decrement in mortality from better emphasis on EMS system in low- and middle-income countries highlight the need to further strengthen the prehospital care and optimization of the number of trained EMTs and paramedic personnel in India [1].

Providing care within a moving ambulance is challenging and depends heavily on the design of the patient cabin — the paramedic’s “working cell” [2]. Confined workspace, suboptimal seating, limited equipment accessibility, and unrestrained equipment compromise provider safety and the ability to deliver effective care, and existing evidence suggests that standard ambulance interiors remain suboptimal when assessed against human factors principles [2–4].

As demand for prehospital services has grown, so has the injury burden among EMS personnel, commonly linked to awkward posture, heavy lifting, forceful extrication, and performing procedures in transit [4]. Motor vehicle collisions and needle-stick injuries remain prominent occupational risks, underscoring the need for targeted ergonomic interventions [5].

Despite the growing importance of prehospital care, limited evidence exists regarding the ergonomic challenges faced by EMS personnel in Indian ambulance settings. Understanding these challenges is essential for improving provider safety, comfort, and patient care during transport. Therefore, this study aimed to evaluate the perspectives of EMTs and paramedics regarding ambulance ergonomics, identify factors affecting safety and comfort during patient transport, and assess occupational injuries and patient safety concerns associated with current ambulance interior design.

## Methods

### Study design and setting

A multicenter cross-sectional survey-based observational study was conducted among Emergency Medical Technologists (EMTs) and paramedics working in prehospital settings across Southern India. Ethical approval was obtained from the Institutional Ethics Committee of Kasturba Medical College, Manipal (IEC2: 382/2023). Following ethics approval, the study was prospectively registered with the Clinical Trials Registry–India (CTRI/2023/10/059357) prior to participant enrolment. Participants were recruited from institutions offering Emergency Medical Technology programs and hospitals employing EMTs involved in prehospital care and patient transport. Institutional permissions were obtained before questionnaire dissemination. Eligible participants were recruited using a convenience sampling approach across the participating centres. Data collection took place between [29/10/2023] and [28/02/2024].

### Questionnaire development

The questionnaire framework was developed after a review of published literature related to ambulance ergonomics, safety, and comfort, including studies by Byran and Gilad and by Ferreira and Hignett. This framework was modified through a focused-group discussion with a panel of 6 Emergency Medical Technologists to assess clarity and relevance to the prehospital context. Content validity was then established by 4 subject experts having at least 7 years of experience in prehospital and emergency medicine care, who reviewed the items for relevance and comprehensiveness. The final instrument consisted of 10 items excluding demographic information and explored ergonomic challenges, safety concerns, occupational injuries, and factors influencing patient care during ambulance transport. Items were organised across four domains — safety and comfort during transit, seat and restraint use, occupational injury, and patient-safety/occupational-health concerns — and used a mix of single-response (e.g., seat-restraint frequency) and multiple-response formats (e.g., factors affecting safety and comfort, injury mechanisms), with multiple-response items explicitly identified as such to respondents. A separate formal pilot test was not conducted; instead, item clarity, relevance, and completion feasibility were assessed through the focused-group discussion with the panel of 6 Emergency Medical Technologists described above, alongside the content-validity review by the 4 subject experts. No formal psychometric pilot (e.g., test-retest reliability or a dedicated pilot sample) was performed, and this is acknowledged as a limitation below. The questionnaire was administered in English via an online survey platform (Google Forms) as well as in paper form for onsite administration, and duplicate submissions were minimised by restricting one response per email address/device and cross-checking respondent identifiers at data cleaning.

### Participants

Eligible participants included Emergency Medical Technicians (EMTs) and Paramedics aged 18 years or older who were actively employed in Emergency Medical Services (EMS) or other prehospital care settings, had prior field experience in prehospital emergency care, and were routinely involved in patient transport using standard Advanced Life Support (ALS) ambulances.

For the purpose of this study, EMTs and Paramedics were defined as prehospital healthcare professionals who had successfully completed a recognized Emergency Medical Technology or Paramedicine education programme (certificate, diploma, or bachelor’s degree) and were qualified to perform emergency patient assessment, stabilization, treatment, and transport within their respective scope of practice. This definition was adopted to encompass the diversity of EMS personnel engaged in ALS ambulance operations while ensuring that all participants possessed formal training and relevant clinical experience.

Participation was voluntary, and informed consent was obtained from all participants before data collection, either electronically for the online survey or in person for the paper-based survey. Data were collected using both online and offline modes to maximize participation and ensure representation of EMS personnel across different operational settings. By including both EMTs and Paramedics with varying levels of professional training and scope of practice, the study aimed to capture a comprehensive range of perspectives on ambulance ergonomics, safety, comfort, and design among personnel directly involved in ALS ambulance-based patient transport.

### Sample size

The minimum required sample size was estimated using a 95% confidence level, an assumed proportion of 70%, a margin of error of 5%, and a target population of 521 EMS personnel, yielding a required sample size of 199 participants.


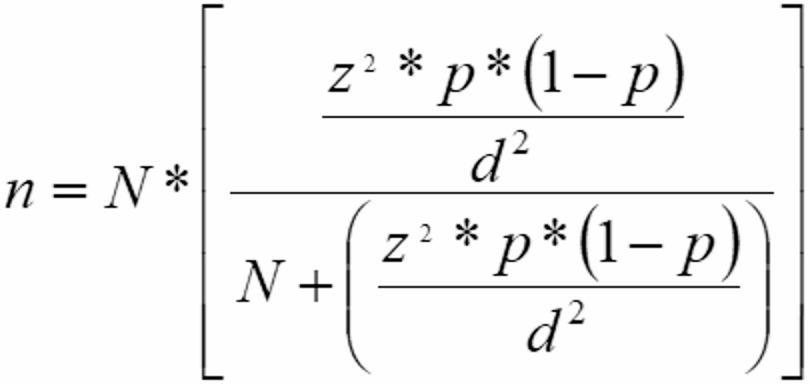


Recruitment was conducted across participating centres during the predefined study period. Participant enrolment was limited by the availability of eligible EMS personnel, institutional participation, and non-response despite repeated dissemination of the survey. A total of 130 complete responses were obtained and included in the final analysis. To assess the adequacy of the achieved sample, the precision of the estimates was evaluated: at 95% confidence, a sample of 130 estimates proportions to within approximately ± 7% points, which is considered adequate for the descriptive, exploratory aims of the study. While the calculated target was not fully achieved, the obtained sample provided an acceptable level of precision for exploratory analysis and reflects the practical challenges of recruiting prehospital EMS personnel in this setting. Enrolment was constrained primarily by the limited size of the eligible population, variable institutional participation, and non-response, and the resulting shortfall from the calculated target of 199 is acknowledged as a limitation below. Non-responders (individuals who accessed but did not complete the questionnaire) were excluded listwise from the final analytic sample, and no imputation was performed for missing responses.

### Statistical analysis

Data were analyzed using jamovi version 2.3.28. Continuous variables were summarized as median and interquartile range (IQR), and categorical variables were presented as frequencies and percentages. Chi-square tests with cross-tabulation were used to examine associations between socio-demographic characteristics and ambulance ergonomics-related variables affecting provider safety, comfort, occupational injuries, and patient care. A p-value of < 0.05 was considered statistically significant.

For the chi-square analyses, age and work experience were dichotomized using the sample median as the cut-off point (age: ≤29 years vs. >29 years; work experience: ≤4 years vs. >4 years). The assumptions of the chi-square test were assessed by reviewing expected cell counts for each cross-tabulation; when this assumption was not fully met, the results were interpreted with caution.

Several questionnaire items allowed multiple responses. For these items, each response option was analyzed separately as a binary variable in relation to age and work experience, rather than being treated as a single categorical variable. Therefore, the p-value reported for each row in Tables 2, 3, 4, 5 and 6 corresponds to the chi-square test for that specific response option against the relevant demographic variable, and not to the item as a whole.

Given the exploratory nature of the study and the absence of complete cross-tabulated output for all comparisons, effect sizes were not reported for every association. Accordingly, the results are presented as exploratory findings intended to generate hypotheses for future research.

## Results

A total of 130 EMTs/paramedics participated in the study. The median age of participants was 29 years (IQR: 7), and 107 (82.3%) were male. The median work experience was 4 years (IQR: 8) (Table 1).

Limited patient-cabin space (78, 60%) and difficulty accessing equipment (65, 50%) were the most frequently reported factors affecting safety and comfort during ambulance transport. While performing procedures in a moving ambulance, 65 (50%) respondents reported concerns related to EMT seat positioning, followed by stretcher-seat orientation (64, 49.23%) and height differences between the stretcher and EMT seat (48, 36.92%) (Table 2).

The use of seat restraints during ambulance transport varied among participants. The largest proportion (54, 41.53%) reported using restraints routinely except during procedures, while 33 (25.38%) reported always using restraints (Table 3).

Uneven roads (64, 49.23%) and sudden vehicle breakdowns (54, 41.53%) were the most frequently reported mechanisms contributing to injuries during emergency responses. Injuries during lifting, shifting, and patient positioning were reported by 66 (50.76%) participants, while 48 (36.92%) reported injuries associated with aggressive patients or bystanders. Cardiopulmonary resuscitation was the procedure most commonly associated with injuries (70, 53.84%), followed by intravenous procedures (56, 43.07%) (Table 4).

Regarding patient safety during transport, intravenous line displacement (58, 44.61%) and difficulty positioning patients on stretchers (58, 44.61%) were the most frequently reported concerns, followed by falls from stretchers (54, 41.53%) (Table 5).

Back pain was the most commonly reported occupational health hazard (75, 57.69%), followed by stress (64, 49.23%), neck pain (42, 32.30%), and psychological trauma (45, 34.61%) (Table 6).

Significant associations were observed between work experience and perceptions regarding EMT seat positioning (*p* = 0.003), injury mechanisms (*p* = 0.002), patient safety concerns (*p* = 0.035), and chronic occupational health hazards (*p* < 0.001).

**Table 1:** Demographic characteristics

Characteristics	Median (IQR) (*n* = 130)
Age (years)	29 (7)
Gender (Male: Female)	107:23
Work experience (years)	4 (8)

**Table 2:** Factors contributing to safety and comfort of a paramedic in an ambulance

Characteristics	Frequency (*n* = 130)	Work Experience (*p* value)	Age (*p* value)
**During transit (without patient)**			
Height of the EMT seat	58 (44.6%)	*p* = 0.708	*p* = 0.999
Cushioning of the seat	34 (26.2%)		
Adjustable seat	47 (36.2%)		
Seat restraints	37 (28.5%)		
Accessibility to equipment	65 (50.0%)		
Total space availability in the patient cabin	78 (60.0%)		
**While performing procedures in a moving ambulance**			
Position of the EMT seat	65 (50.0%)	*p* = 0.003	*p* = 0.768
Height difference between stretcher and seat	48 (36.9%)		
Orientation of seating position/posture to patient	64 (49.2%)		
Accessibility to equipment	57 (43.8%)		
Speed of the vehicle	40 (30.8%)		
Availability of PPE/gown	48 (36.9%)		

**Table 3:** Use of seat restraints in the ambulance

Characteristics	Frequency (*n* = 130)	Work Experience (*p* value)	Age (*p* value)
Not available	13 (10.0%)	*p* = 0.422	*p* = 0.712
Always	33 (25.4%)		
Always, except during procedures	54 (41.5%)		
Sometimes	26 (20.0%)		
Never	10 (7.7%)		

**Table 4:** Injuries to care providers while in the ambulance

Characteristics	Frequency (*n* = 130)	Work Experience (*p* value)	Age (*p* value)
**Mechanism causing injury**			
Road traffic accidents	44 (33.8%)	*p* = 0.002	*p* = 0.486
Sudden breakdown	54 (41.5%)		
Uneven roads	64 (49.2%)		
Injury from not using restraints	33 (25.4%)		
Projectile injury from non-secured equipment	36 (27.7%)		
**Occupational injury during emergency response**			
Road traffic accidents involving ambulance	44 (33.8%)	*p* = 0.108	*p* = 0.260
Needle-stick injuries	51 (39.2%)		
Exposure to blood/body fluid splash	45 (34.6%)		
Violent/aggressive patient or attendant	48 (36.9%)		
Injury during lifting and patient positioning	66 (50.8%)		
Tripping over tubing or wires	15 (11.5%)		
**Injuries while performing procedures**			
Cardiopulmonary resuscitation	70 (53.8%)	*p* = 0.111	*p* = 0.101
Airway maneuvers	38 (29.2%)		
Oxygen therapy	26 (20.0%)		
Haemorrhage control	39 (30.0%)		
IV cannulation / IO / connecting IV set	56 (43.1%)		
Splinting, fractures, penetrating injuries	48 (36.9%)		

**Table 5:** Safety concerns to patients during ambulance transfer

Characteristics	Frequency (*n* = 130)	Work Experience (*p* value)	Age (*p* value)
Falls	54 (41.5%)	*p* = 0.035	*p* = 0.888
Projectile injuries from equipment	42 (32.3%)		
Collisional trauma	50 (38.5%)		
Intravenous line displacement	58 (44.6%)		
Endotracheal tube dislodgement/disconnection	23 (17.7%)		
Difficult positioning on stretcher	58 (44.6%)		

**Table 6:** Chronic occupational health hazards associated with frequent ambulance transport

Characteristics	Frequency (*n* = 130)	Work Experience (*p* value)	Age (*p* value)
Back pain	75 (57.7%)	*p* < 0.001	*p* < 0.001
Neck pain	42 (32.3%)		
Sprains/fractures	25 (19.2%)		
Stress	64 (49.2%)		
Depression/psychological trauma	45 (34.6%)		
Chemical exposure	5 (3.8%)		

## Discussion

**Fig. 1:**
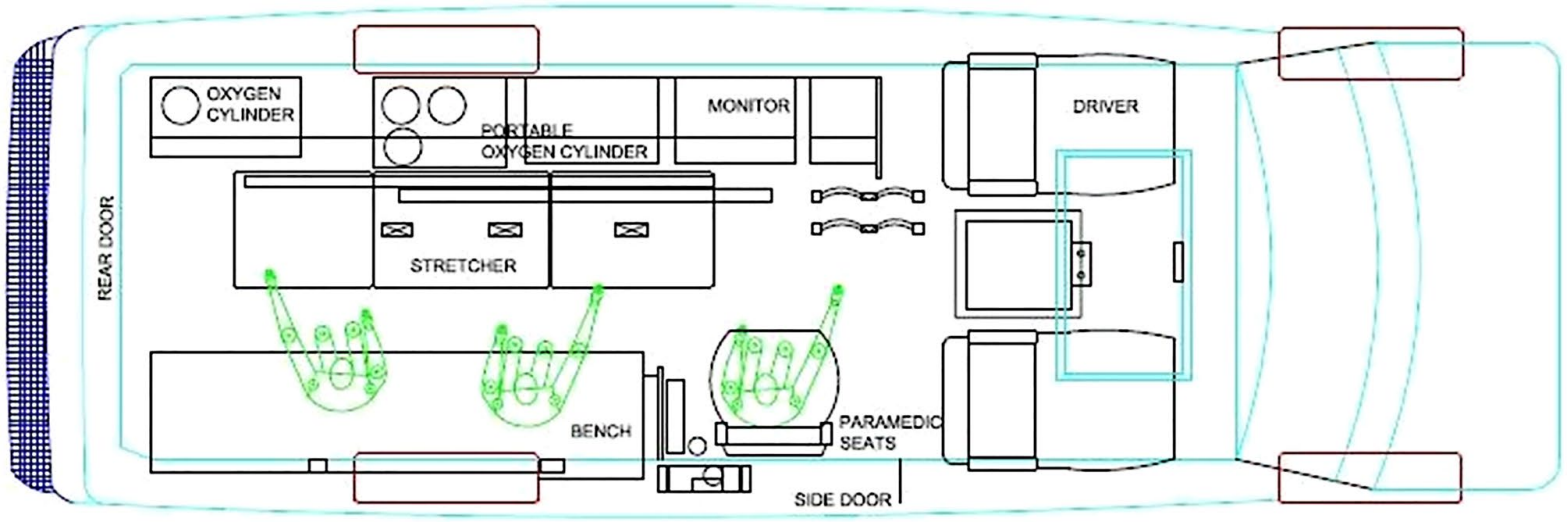
Representative of standard ALS ambulance using ZW CAD – CAD model

This study examined EMT and paramedic perspectives on ambulance ergonomic design, using the standard ALS ambulance configuration shown in Fig. 1 as the reference layout, and identified several areas of concern relevant to occupational safety, comfort, and patient care during transport.

**Fig. 2:**
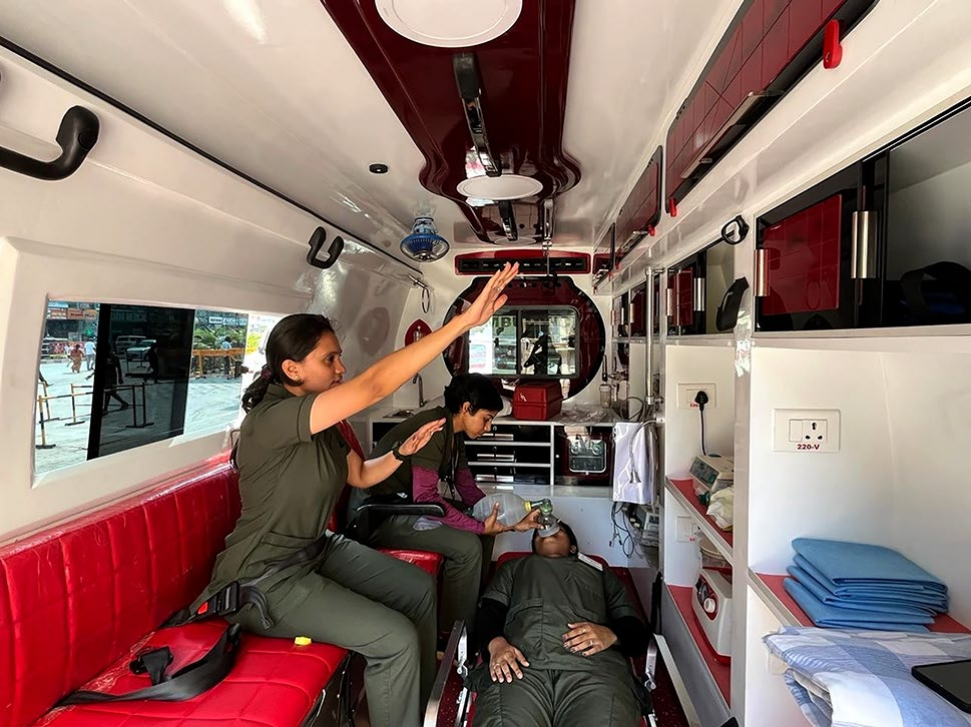
Accessibility of equipment from the side seat / bench

**Fig. 3:**
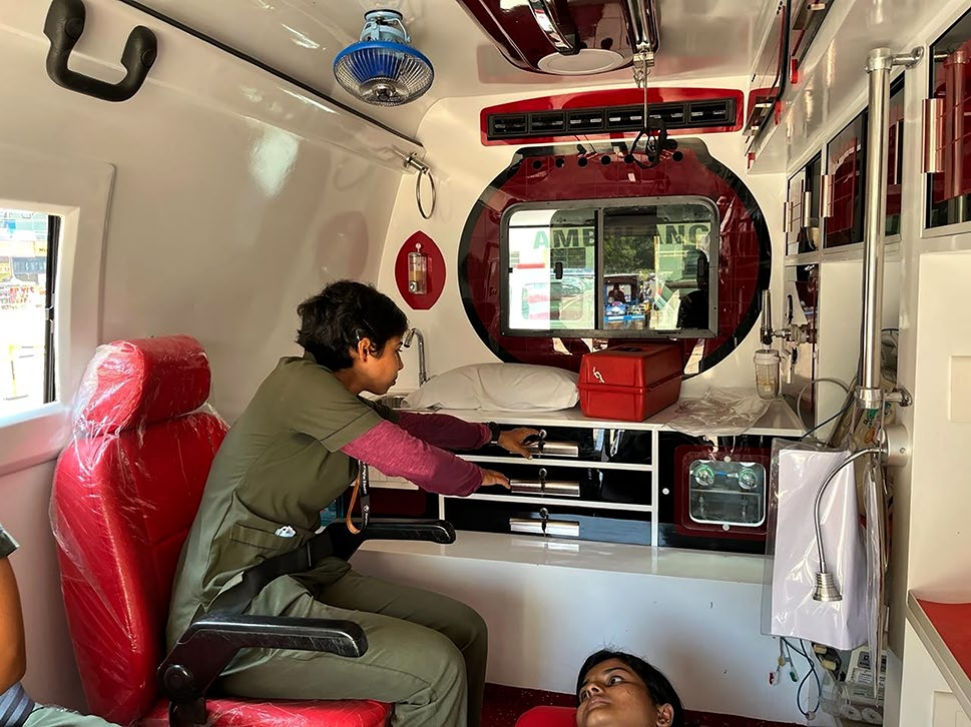
Position of the EMT seat

Seat position and equipment access were consistently identified as concerns. These findings align with earlier ergonomic evaluations reporting that paramedics frequently find EMT seat positioning inefficient for performing procedures and side benches uncomfortable [6, 7]. In the present study, 58 respondents (44.6%) reported that the EMT seat is positioned higher than the patient stretcher, requiring extensive bending and prolonged standing; this is consistent with earlier reports of paramedics adopting non-neutral back postures exceeding 20° during patient care [6, 7]. Sixty-five respondents (50%) also reported difficulty accessing wall-mounted and compartment-stored equipment from both the paramedic seat and the side bench (Fig. 2), and a further 65 (50%) were dissatisfied with EMT seat position itself (Fig. 3), citing restricted access to the patient’s left side and to devices such as ventilators and defibrillators. Together, these findings directly informed the seat-repositioning concept illustrated in Fig. 5, although this remains a preliminary design direction requiring formal ergonomic testing rather than a validated solution.

Limited total cabin workspace was reported by 78 respondents (60%) and was described as particularly restrictive during long-distance transfers and procedures such as CPR that require prolonged standing. This concern is reflected in the expanded-workspace concept shown in Fig. 7. Seat-restraint use was similarly constrained by the physical demands of patient care: the largest group of respondents, 54 (41.53%), reported using restraints routinely except during procedures, suggesting that current restraint design creates a trade-off between occupant safety and the practical requirements of delivering care [8, 9].

Beyond interior layout, ambulance road crashes were identified as a further source of risk. Uneven roads (64, 49.23%) and sudden vehicle breakdown (54, 41.53%) were the most frequently cited contributing factors in this study, consistent with broader literature describing ambulance crashes and their associated injury and fatality burden among EMS personnel [10–14]. The absence of seat restraints and the presence of unsecured equipment within the patient cabin may further increase injury risk during a crash, although this study did not directly measure crash outcomes.

**Fig. 4:**
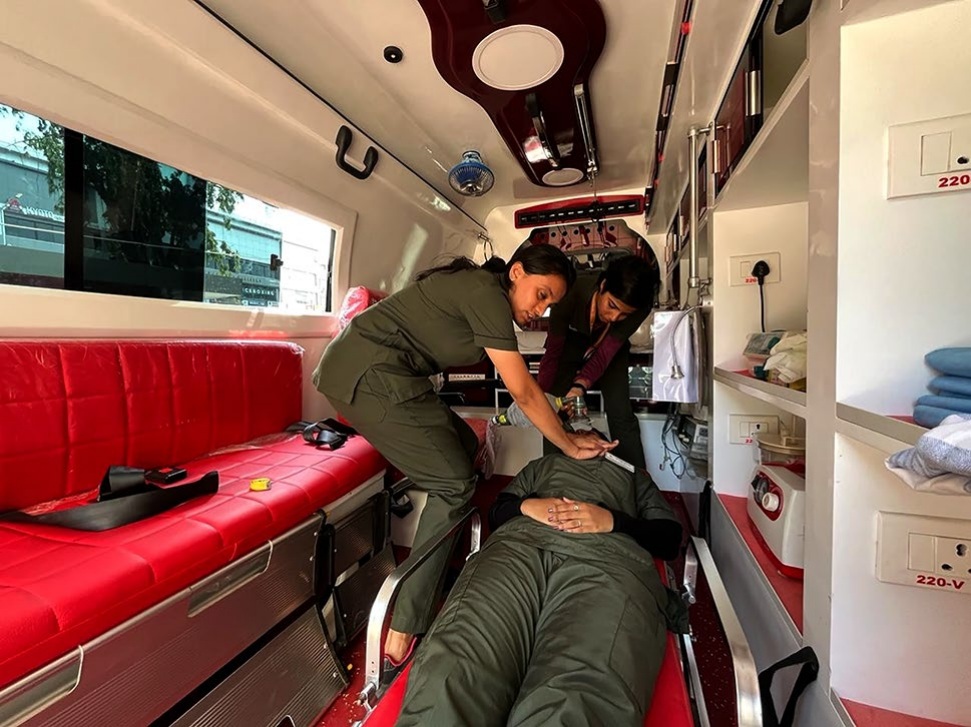
Performing CPR in ambulance

Procedural care added further risk: CPR performed in a moving ambulance was the procedure most frequently associated with injury (70, 53.84%), reflecting the limited workspace noted above (Fig. 4). Simulation-based research using virtual ambulance driving platforms has previously been used to evaluate how vehicle motion affects CPR quality, and a similar approach could help quantify this risk more precisely than self-reported perception alone [15]. A further 56 respondents (43.07%) reported susceptibility to needle-stick injury during intravenous cannulation, consistent with occupational injury patterns reported elsewhere among EMS personnel [5, 16].

Beyond physical injury, respondents reported chronic occupational health concerns, including back pain (75, 57.69%) and stress (64, 49.23%), consistent with the demanding physical and psychological nature of EMS work [17]. Patient safety during transfer was also raised as a concern, with 54 respondents (41.53%) identifying stretcher falls, attributed to the absence of stretcher restraints, as the most frequently observed incident; similar transport-related incidents have been reported in other out-of-hospital settings [18].

Work experience was significantly associated with perceptions of EMT seat positioning (*p* = 0.003), injury mechanisms (*p* = 0.002), patient safety concerns (*p* = 0.035), and occupational health hazards (*p* < 0.001). These associations were not stratified by experience group in the present analysis.

These associations suggest that work experience may shape how EMS personnel perceive ambulance seating, injury risk, patient safety, and chronic occupational strain. Because this was a cross-sectional survey based on self-reported perceptions, the findings should be interpreted as exploratory.

**Fig. 5:**
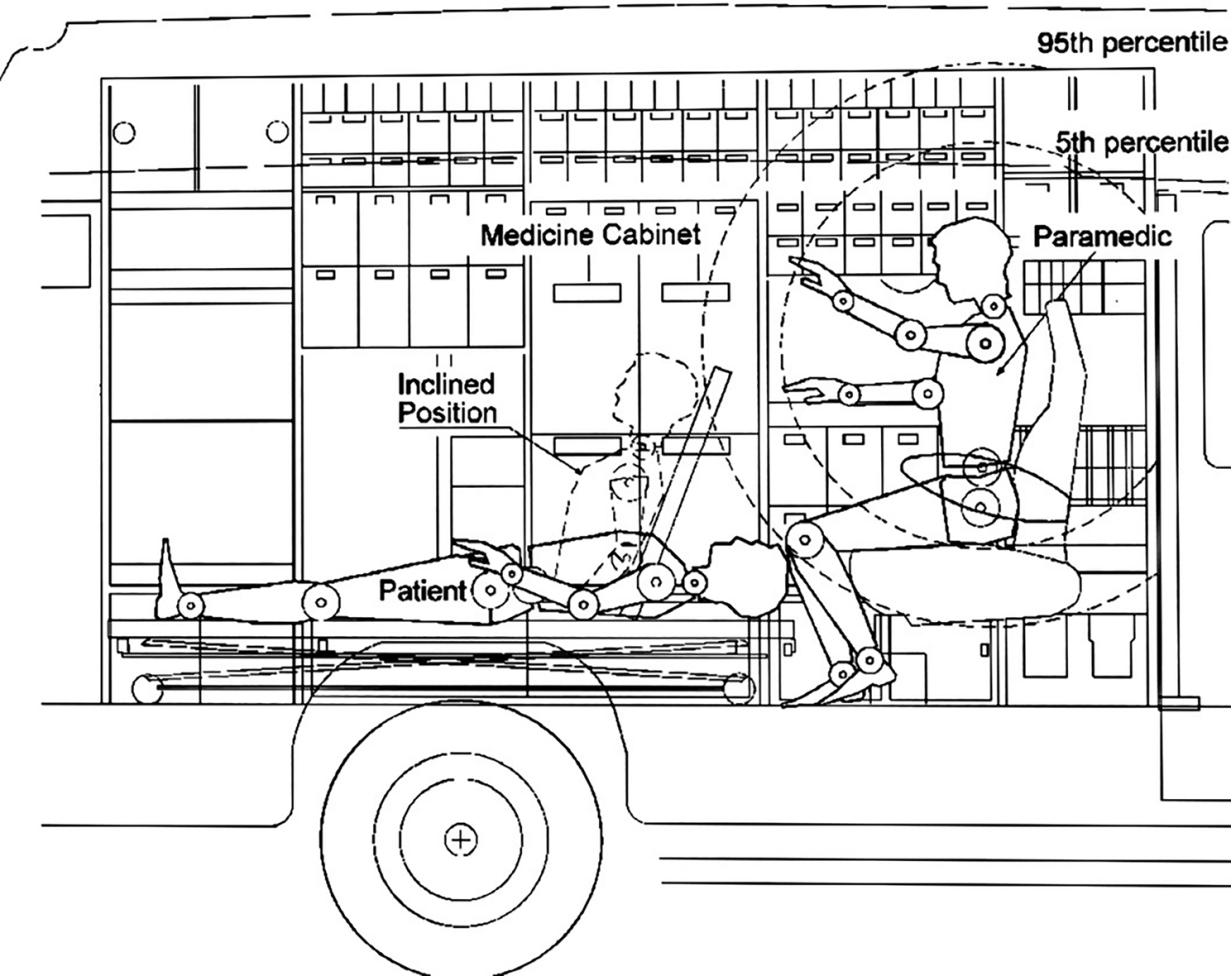
Preliminary ergonomic concept 1

**Fig. 6:**
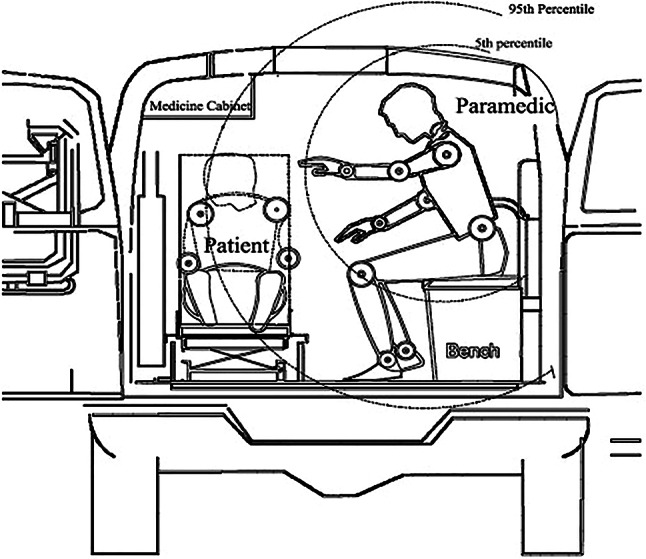
Preliminary ergonomic concept 2

**Fig. 7:**
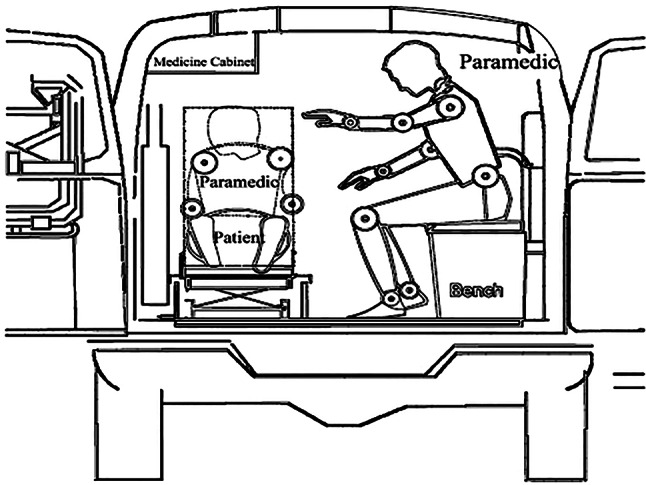
Preliminary ergonomic concept 3

Overall, these findings describe consistent, perception-based concerns regarding ambulance ergonomic design, restraint use, and occupational safety among EMS personnel in this setting. They point to specific, testable directions for design and system-level improvement, illustrated in Figs. 5, 6 and 7, but as a survey-based study they identify problems and plausible directions for solutions rather than validating them; the design concepts presented here require further ergonomic, engineering, and simulation-based evaluation before implementation.

## Recommendations

Design modification was not an objective of this study, and the points below are not validated ambulance design recommendations. They represent directions for future research that follow from participants’ reported perceptions and existing ergonomic literature [9, 19, 20], and would require formal ergonomic evaluation, engineering assessment, and simulation-based testing before any design change could be considered evidence-based.

- Further research into EMT seat orientation and position, informed by the concerns reported by participants and illustrated as preliminary concepts in Figs. 5, 6 and 7, is needed before any modification is implemented.
- Ambulance-based simulation studies, similar to those used to evaluate CPR quality under different vehicle-motion conditions [15], combined with structured questionnaires, interviews, or focus group discussions, are recommended to characterise safety, comfort, and injury risk with greater precision than self-reported perception alone.
- Further research into driver training on road safety and speed control, and into occupational health and posture-awareness programmes for EMS personnel, building on established human-factors design frameworks [20], is recommended.

Half of the paramedics (50%) reported problems with the position of the EMT seat; repositioning the EMT seat may improve access to equipment and the medicine cabin and support patient care.

Understanding the concerns raised by the paramedic related to the total working space available, this design represents increased space in the patient cabin, making it comfortable to provide care.

## Conclusions

EMS is a developing field, and the future evolution of paramedic practice and prehospital transport is an area of growing attention [21]. This survey-based study describes EMT and paramedic perceptions of ambulance ergonomics, safety, and occupational health, and identifies EMT seat position, equipment accessibility, cabin workspace, and restraint design as recurring areas of concern. These findings represent self-reported perceptions rather than validated ergonomic or design conclusions and should be interpreted as a basis for further objective research rather than as evidence supporting specific design changes.

## Limitation

This study has several limitations that should be considered when interpreting the findings. As a survey-based analysis of individual perspectives, the results reflect participants’ subjective perceptions rather than objective ergonomic or biomechanical measurement; no direct assessment of posture, workspace dimensions, or physical strain was performed. Self-administered questionnaires are also subject to self-report and recall bias, including socially desirable responding and inaccurate recollection of past events. The questionnaire’s content was reviewed through a focused-group discussion and expert content-validity review rather than a separate formal pilot test, and no psychometric validation (e.g., test-retest reliability or construct validity) was undertaken; this represents an area for future methodological development. Participants were recruited through convenience sampling across participating centres, which may have introduced selection bias, and the achieved sample of 130 fell short of the calculated target of 199, reflecting the practical difficulty of recruiting EMS personnel in this setting; the shortfall may limit precision and generalizability. The cross-sectional design captures perceptions at a single time point and cannot establish causal or temporal relationships between ambulance ergonomics and reported injury. Findings are based on a multicenter sample from Southern India and may not generalize to other regions, ambulance configurations, or EMS systems. Finally, this study did not set out to design or validate ambulance modifications, and no biomechanical or engineering validation of the design concepts discussed was undertaken.

## Data Availability

No datasets were generated or analysed during the current study.
